# Supplementary material for: Therapeutic potential of a choline-zinc-vitamin E nutraceutical complex in ameliorating thioacetamide-induced nonalcoholic fatty liver pathology in zebrafish
Source: PLoS One. 2025 May 20;20(5):e0324164. doi: 10.1371/journal.pone.0324164 (PMC12091810; doi:10.1371/journal.pone.0324164)
Supplement: S1 File — S1 Fig. Wayne diagrams of differential genes. CB: Choline bitartrate control group; FF: Nutraceutical complex containing Zinc, Choline bitartrate, and Vitamin E. S2 Fig. GO annotation analysis of the choline bitartrate differential gene. (A) GO classification of the choline bitartrate differential gene. (B) GO Biological process enrichment bubble diagram. (C) GO Molecular function enrichment bubble diagram. (D) GO Cellular component enrichment bubble map. S3 Fig. GO annotation analysis of functional formulation differential genes. (A) GO classification of the Functional formulation differential gene. (B) GO Biological process enrichment bubble diagram. (C) GO Cellular component enrichment bubble map. (D) GO Molecular function enrichment bubble diagram. S4 Fig. Differential gene KEGG enrichment. (A) KEGG enrichment of the choline bitartrate differential gene. (B) KEGG enrichment of the functional formulation differential gene. S1 Table. The concentration of nutraceutical complex for the protective effects against non-alcoholic fatty liver. S2 Table. The protective effect of nutraceutical complex on NAFLD (alanine transaminase activity). S3 Table. The protective effect of nutraceutical complex on NAFLD (Aspartate aminotransferase activity). S4 Table. The pathways and differential genes involved in the nutraceutical complex intervention group. S5 Table. Effect of nutraceutical complex on the expression of NAFLD-associated proteins (n = 3). S6 Table. Results of the acsl1a-mRNA dose mapping experiment (n = 30). S7 Table. Concentration of total RNA and A260/A280 ratio (n = 3). S8 Table. Primer Sequence Information. S9 Table. Results of experiments to evaluate the protective efficacy of samples against NAFLD (n = 10). (DOCX) [file pone.0324164.s001.docx]

**S1 Fig. Wayne diagrams of differential genes. CB: Choline bitartrate control group; FF: Nutraceutical complex containing Zinc, Choline bitartrate, and Vitamin E.**


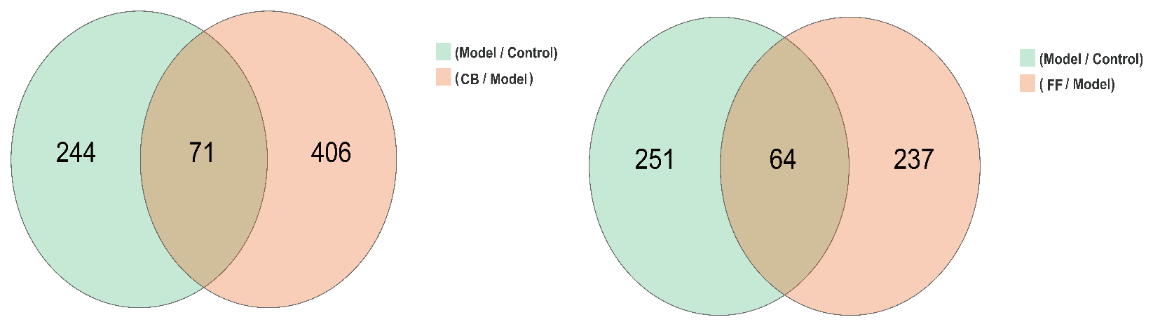


**S2 Fig. GO annotation analysis of the choline bitartrate differential gene. (A) GO classification of the choline bitartrate differential gene. (B) GO Biological process enrichment bubble diagram. (C) GO Molecular function enrichment bubble diagram. (D) GO Cellular component enrichment bubble map.**


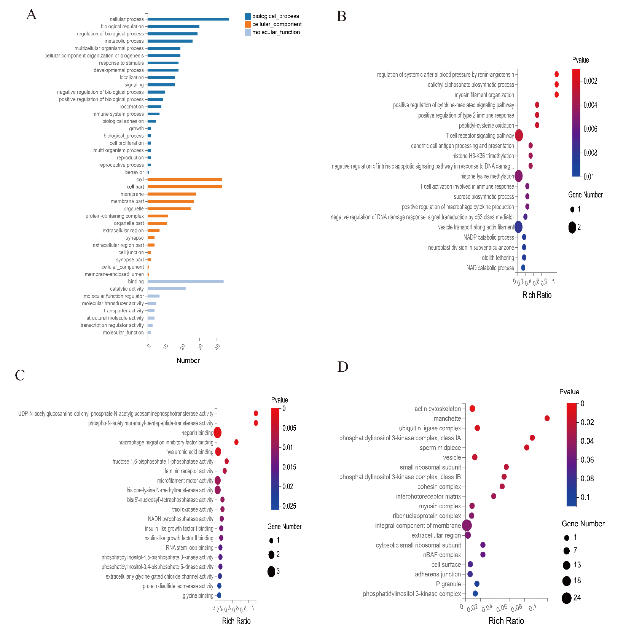


**S3 Fig. GO annotation analysis of functional formulation differential genes. (A) GO classification of the Functional formulation differential gene. (B) GO Biological process enrichment bubble diagram. (C) GO Cellular component enrichment bubble map. (D) GO Molecular function enrichment bubble diagram.**


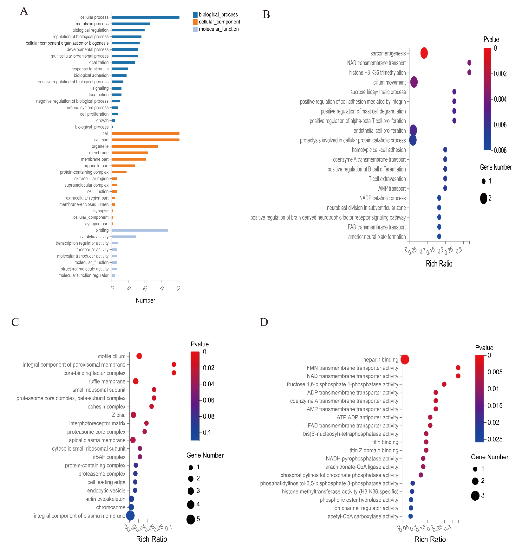


**S4 Fig. Differential gene KEGG enrichment. (A) KEGG enrichment of the choline bitartrate differential gene. (B) KEGG enrichment of the functional formulation differential gene.**


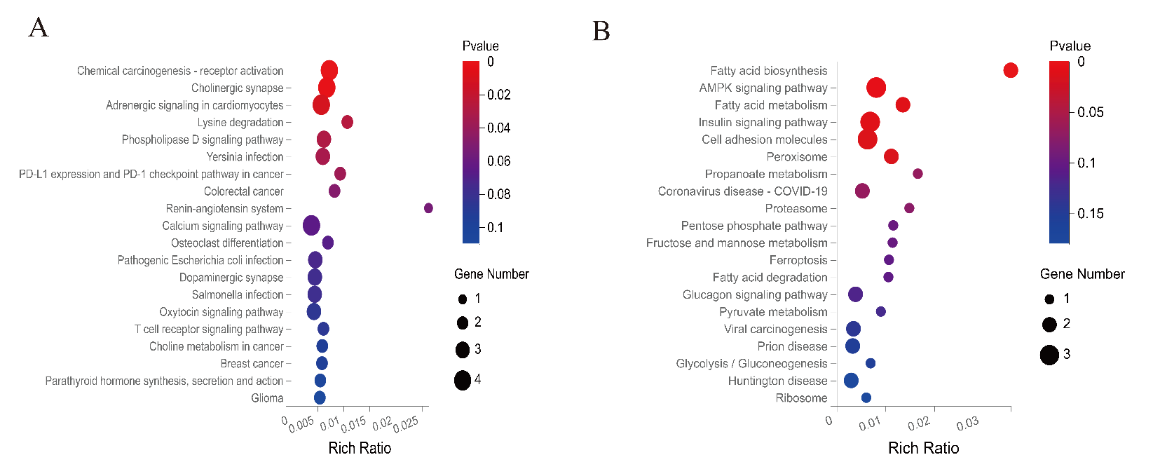


S1 Table. The concentration of nutraceutical complex for the protective effects against non-alcoholic fatty liver.

| Group | concentration（µg/mL） | Number of death（well） | Morality rate（%） | Phenotype |
| --- | --- | --- | --- | --- |
| Normal control group (n=30) | - | 0 | 0 | No apparent abnormalities |
| Model control group(n=30) | - | 0 | 0 | No apparent abnormalities |
| Nutraceutical complex containing zinc, choline bitartrate, and vitamin E (n=30) | 125 | 0 | 0 | Like the model control group |
|  | 250 | 0 | 0 | Like the model control group |
|  | 500 | 0 | 0 | Like the model control group |
|  | 1000 | 6 | 20 | - |
|  | 2000 | 24 | 80 | - |

**S2 Table. The protective effect of nutraceutical complex on NAFLD (alanine transaminase activity).**

| Group | concentration（µg/mL） | alanine transaminase activity in the tissue（U/ g protein，mean ± SE） |
| --- | --- | --- |
| Normal control group(n=3) | - | 17.8 ± 0.232^a^ |
| Model control group(n=3) | - | 24.5 ± 0.331 |
| Positive control group(n=3) | 50.0 | 18.4 ± 0.324 ^a^ |
| Nutraceutical complex containing zinc, choline bitartrate, and vitamin E (n=3) | 125 | 23.7 ± 0.414 |
|  | 250 | 21.5 ± 0.101 ^a^ |
|  | 500 | 19.1 ± 0.314 ^a^ |
| Choline bitartrate (n=3) | 81.0 | 23.2 ± 0.525 |
|  | 162 | 23.2 ± 0.769 |
|  | 316 | 21.3 ± 0.910 ^a^ |

^a^ Compared with the control group of the model, *p* < 0.05.

**S3 Table. The protective effect of nutraceutical complex on NAFLD (Aspartate aminotransferase activity).**

| Group | concentration（µg/mL） | aspartate aminotransferase activity in the tissue（U/ g protein，mean ± SE） |
| --- | --- | --- |
| Normal control group  (n=3) | - | 21.1 ± 0.371 ^a^ |
| Model control group(n=3) | - | 32.9 ± 0.579 |
| Positive control group(n=3) | 50.0 | 26.3 ± 0.360 ^a^ |
| Nutraceutical complex containing zinc, choline bitartrate, and vitamin E(n=3) | 125 | 31.2 ± 1.50 |
|  | 250 | 27.9 ± 0.206 ^a^ |
|  | 500 | 24.4 ± 0.085 ^a^ |
| Choline bitartrate(n=3) | 81.0 | 30.7 ± 1.66 |
|  | 162 | 30.7± 0.392 |
|  | 316 | 28.8± 0.430 ^a^ |

^a^ Compared with the control group of the model, *p* < 0.05.

**S4 Table. The pathways and differential genes involved in the nutraceutical complex intervention group.**

| pathways | P value | genes |
| --- | --- | --- |
| Fatty acid biosynthesis | < 0.01 | *acsl1a、acaca* |
| AMPK signaling pathway | < 0.01 | *fbp2、tbc1d1、acaca* |
| Fatty acid metabolism | < 0.01 | *acsl1a、acaca* |
| fructose 1,6-bisphosphate 1-phosphatase activity | < 0.01 | *fbp2* |

**S5 Table. Effect of nutraceutical complex on the expression of NAFLD-associated proteins (n = 3).**

| Group | concentration （μg/mL） | Relative expression of ACACA protein（mean ± SE） | Relative expression of TBC1D1 protein （mean ± SE） | Relative expression of ACSL1 protein （mean ± SE） | Relative expression of FBP2 protein （mean ± SE） |
| --- | --- | --- | --- | --- | --- |
| Normal control group | - | 0.541 ± 0.078** | 1.07 ± 0.127* | 0.344 ± 0.021*** | 0.698 ± 0.058** |
| Model control group | - | 1.14 ± 0.065 | 0.694 ± 0.034 | 0.859 ± 0.055 | 1.07 ± 0.055 |
| Nutraceutical complex containing zinc, choline bitartrate, and vitamin E | 125 | 1.10 ± 0.066 | 0.773 ± 0.018 | 0.814 ± 0.075 | 1.04 ± 0.091 |
|  | 250 | 0.876 ± 0.056* | 0.886 ± 0.018* | 0.551 ± 0.066* | 0.823 ± 0.021* |
|  | 500 | 0.766 ± 0.031** | 0.971 ± 0.073** | 0.441 ± 0.054** | 0.709 ± 0.018** |

Compared with the control group of the model. **p* < 0.05, ***p* < 0.01, ****p* < 0.001.

**S6 Table. Results of the acsl1a-mRNA dose mapping experiment (n = 30).**

| **Group** | **Dose (ng/tail).** | **Deaths (tail)** | **Mortality rate (%)** | **phenotype** |
| --- | --- | --- | --- | --- |
| **Normal control group** | - | 0 | 0 | No significant abnormalities were observed |
| ***acsl1a-*mRNA** | 0.285 | 0 | 0 | Similar status to the normal control group |
|  | 0.571 | 0 | 0 | Similar status to the normal control group |
|  | 1.14 | 10 | 33 | - |
|  | 2.28 | 18 | 60 | - |
|  | 4.57 | 30 | 100 | - |

**S7 Table. Concentration of total RNA and A260/A280 ratio (n = 3).**

| **group** | **dosage**  **(ng/tail).** | **RNA concentration (μg/μL)** | | | **A260/A280** | | |
| --- | --- | --- | --- | --- | --- | --- | --- |
|  |  | **Sample 1** | **Sample 2** | **Sample 3 3three** | **Sample 1** | **Sample 2** | **Sample 3 three** |
| Normal control group | - | 0.644 | 0.646 | 0.499 | 1.94 | 1.97 | 1.95 |
| *acsl1a-*mRNA | 0.571 | 0.497 | 0.665 | 0.663 | 1.95 | 1.91 | 1.93 |

**S8 Table. Primer Sequence Information.**

| **gene** | **Primer sequences** | |
| --- | --- | --- |
| ***β-actin*** | Forward | 5’-TCGAGCAGGAGATGGGAACC-3’ |
|  | Reverse | 5’-CTCGTGGATACCGCAAGATTC-3’ |
| ***acsl1a*** | Forward | 5’-TAACACCACTGAGACGTTGC-3’ |
|  | Reverse | 5’-ATGTGGTGATGGCAGCGAAT-3’ |

**S9 Table. Results of experiments to evaluate the protective efficacy of samples against NAFLD (n = 10).**

| group | concentration（µg/mL） | Liver fat staining intensity  (pixels, mean ± SE). |
| --- | --- | --- |
| Normal control group | - | 6823 ± 417 ^a^ |
| Model control group | - | 10053 ± 252 |
| Nutraceutical complex containing zinc, choline bitartrate, and vitamin E | 125 | 9514 ± 307 |
|  | 250 | 7369 ± 374 ^a^ |
|  | 500 | 7232 ± 275 ^a^ |

^a^ **Compared with the model control group, p < 0.001**

**The original underlying images for all blot**

**
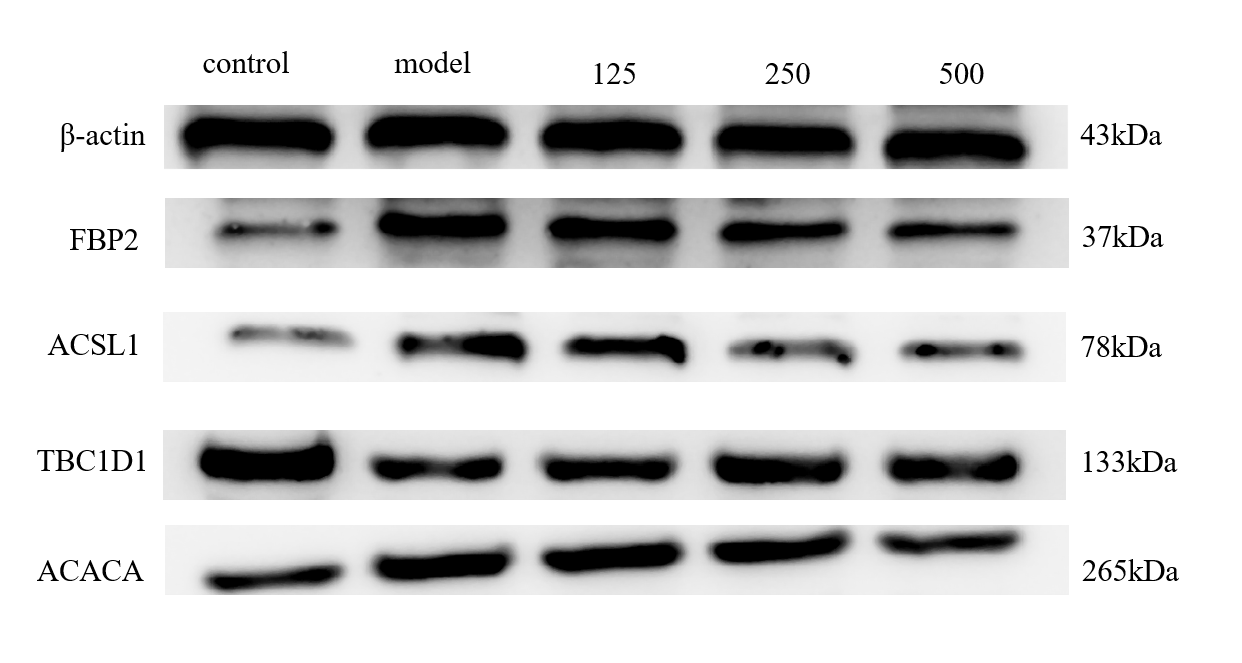
**

**Protein blotting of the first repeat**

**
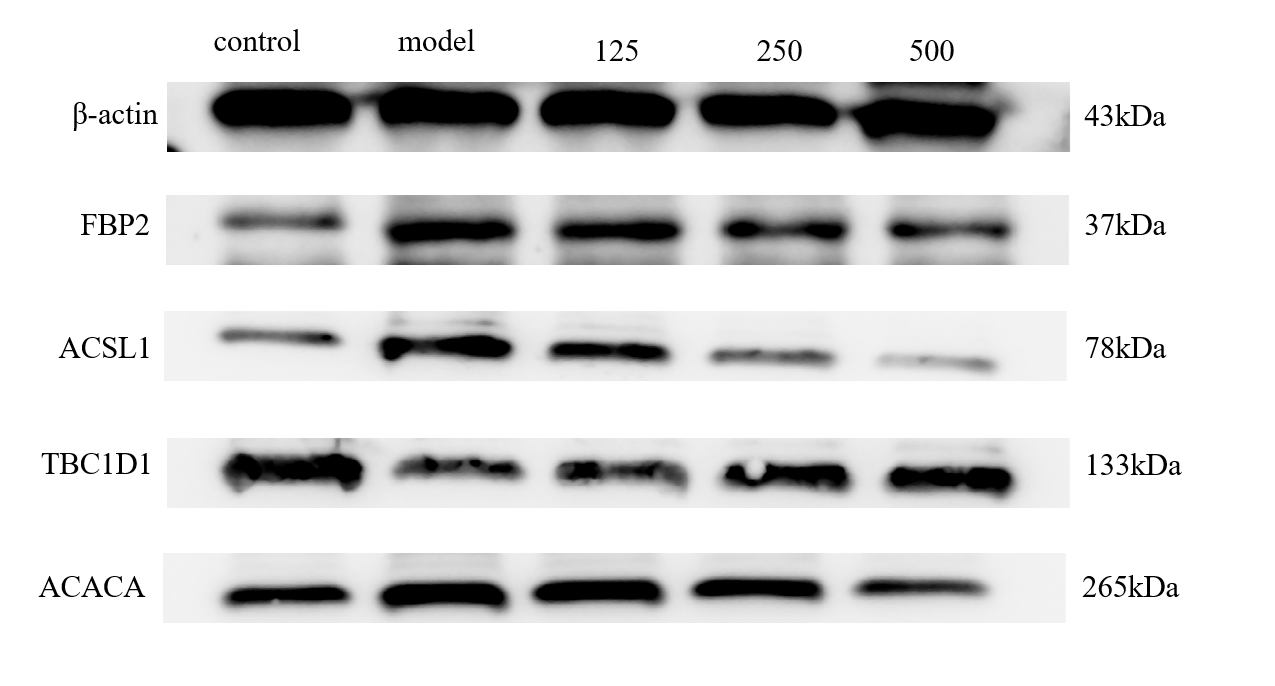
**

**Protein blotting of the second repeat**

**
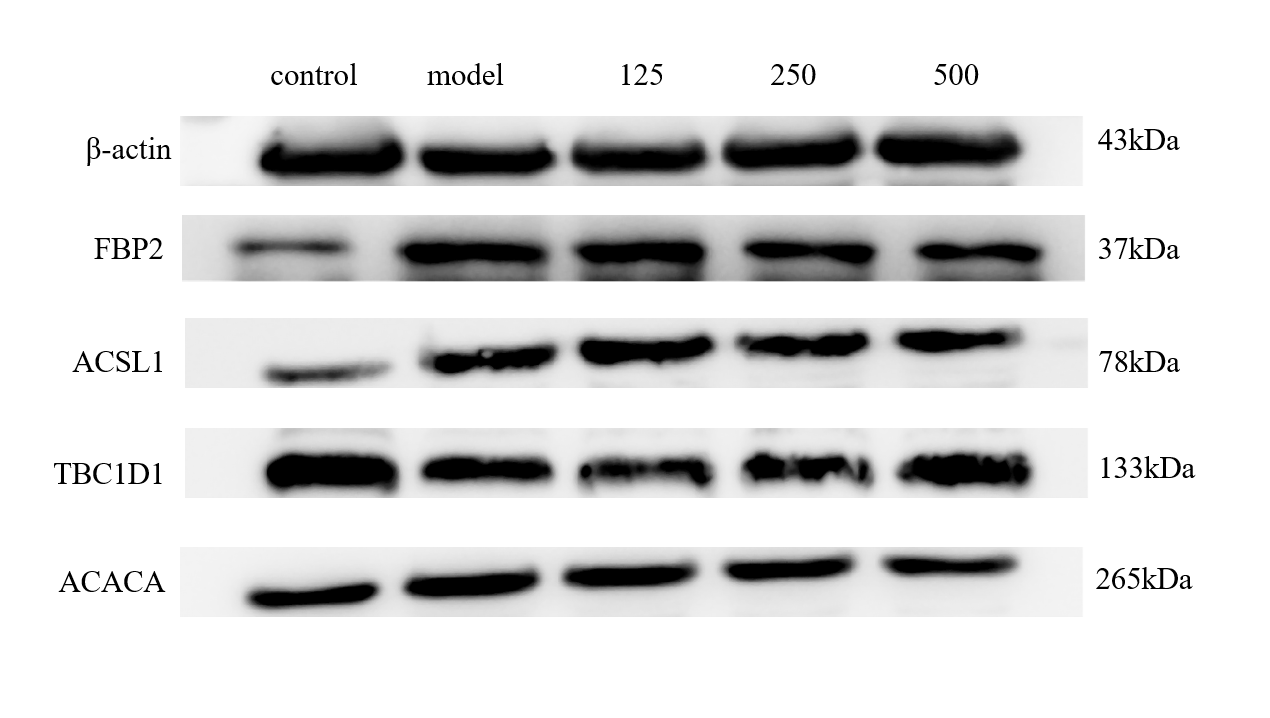
**

**Protein blotting of the third repeat**
