# Supplementary material for: Therapeutic potential of a choline-zinc-vitamin E nutraceutical complex in ameliorating thioacetamide-induced nonalcoholic fatty liver pathology in zebrafish
Source: PLoS One. 2025 May 20;20(5):e0324164. doi: 10.1371/journal.pone.0324164 (PMC12091810; doi:10.1371/journal.pone.0324164)
Supplement: Attachment — Submitted filename: Comment PLOS 1.docx [file pone.0324164.s003.docx]

Comment:

- In “Hematoxylin and Eosin Staining, and Oil Red O Staining” paragraph, kindly mention the full form of H&E.
- In line 213, kindly mention the sample name.
- In result S1, S2, S3, S4 table and Figure means which table and figure? In supporting information no figure and table found. Kindly check.
- In Table 1 , which substrate’s concentration? Please mention.
