# Supplementary material for: Therapeutic potential of a choline-zinc-vitamin E nutraceutical complex in ameliorating thioacetamide-induced nonalcoholic fatty liver pathology in zebrafish
Source: PLoS One. 2025 May 20;20(5):e0324164. doi: 10.1371/journal.pone.0324164 (PMC12091810; doi:10.1371/journal.pone.0324164)
Supplement: Attachment — Submitted filename: Response_to_Reviewers_auresp_2.docx [file pone.0324164.s005.docx]

Dear Editors and Reviewers,

Thank you very much for your work in reviewing this manuscript. We have made careful revisions based on the editor's and reviewer's comments, and we hope that the revised manuscript can satisfy you; the specific responses to the comments are shown in the following responses. All the contents revised this time have been marked with red font.

**Response to “Journal Requirements”:**

1. Please review your reference list to ensure that it is complete and correct. If you have cited papers that have been retracted, please include the rationale for doing so in the manuscript text, or remove these references and replace them with relevant current references. Any changes to the reference list should be mentioned in the rebuttal letter that accompanies your revised manuscript. If you need to cite a retracted article, indicate the article’s retracted status in the References list and also include a citation and full reference for the retraction notice.

**Response: Thank you very much for your reminder. We have checked the references of the manuscript, and all references are correct and complete, and there are no withdrawn references.**

**Response to Reviewer-2**:
1、In “Hematoxylin and Eosin Staining, and Oil Red O Staining” paragraph, kindly mention the full form of H&E.

**Response: Thank you very much for your careful and valuable comments, we have added the full form of H&E in the appropriate section.**

2、In line 213, kindly mention the sample name.

**Response: Thank you very much for your careful comments, we have made changes accordingly. We change “ the MTC of the sample ” to “ the Maximum tolerated concentration (MTC) of the nutraceutical complex containing choline bitartrate, zinc citrate, and dl-α-Tocopheryl acetate ”.**

3、In result S1, S2, S3, S4 table and Figure means which table and figure? In supporting information no figure and table found. Kindly check.

**Response: Thank you for your careful comments, we have re-checked and revised them carefully to ensure that the citations in the body of the text correspond to the content of the supplementary material. In the support information section, we have also added images and tables.**

4、In Table 1 , which substrate’s concentration? Please mention.

**Response: Thank you very much for your comments, we have added superscripts and added instructions below the table as follows:**

**In the positive control group, the concentration represents the concentration of polyene phosphatidylcholine. In addition, the nutraceutical complex group containing zinc, choline bitartrate, and vitamin E and the choline bitartrate group each had three concentration levels.**
